# Ultrasensitive Frequency Shifting of Dielectric Mie Resonance near Metallic Substrate

**DOI:** 10.34133/2022/9862974

**Published:** 2022-05-09

**Authors:** Chuanbao Liu, Changxin Wang, Junhong Chen, Yanjing Su, Lijie Qiao, Ji Zhou, Yang Bai

**Affiliations:** ^1^School of Materials Science and Engineering, University of Science and Technology Beijing, Beijing 100083, China; ^2^Beijing Advanced Innovation Center for Materials Genome Engineering, Institute for Advanced Materials and Technology, University of Science and Technology Beijing, Beijing 100083, China; ^3^State Key Laboratory of New Ceramics and Fine Processing, Tsinghua University, Beijing 100084, China

## Abstract

Dielectric resonators on metallic surface can enhance far-field scattering and boost near-field response having promising applications in nonlinear optics and reflection-type devices. However, the dependence of gap size between dielectric resonator and metallic surface on Mie resonant frequency is complex and desires a comprehensive physical interpretation. Here, we systematically study the effect of metallic substrate on the magnetic dipole (MD) resonant frequency at X-band by placing a high permittivity CaTiO_3_ ceramic block on metallic substrate and regulating their gap size. The simulated and experimental results show that there are two physical mechanisms to codetermine the metallic substrate-induced MD frequency. The greatly enhanced electric field pair in the gap and the coupling of MD resonance with its mirror image are decisive for small and large gaps, respectively, making the MD resonant frequency present an exponential blue shift first and then a slight red shift with increasing gap size. Further, we use the two mechanisms to explain different frequency shifting properties of ceramic sphere near metallic substrate. Finally, taking advantage of the sharp frequency shifting to small gaps, the ceramic block is demonstrated to accurately estimate the thickness or permittivity of thin film on metallic substrate through a governing equation derived from the method of symbolic regression. We believe that our study will help to understand the resonant frequency shifting for dielectric particle near metallic substrate and give some prototypes of ultrasensitive detectors.

## 1. Introduction

In recent years, high-refractive-index dielectric particles have attracted much attention due to their abundant electromagnetic Mie resonances which provide a versatile platform to modulate scattering wave (polarization, amplitude, phase, and wavefront) and boost near-field response, having wide range of applications in biomedical engineering [1, 2], optical components [3–5], and nonlinear optics [6–8]. Typically, for dielectric particles considerably smaller than incident wavelength, the scattering spectrum is dominated by the first two modes of Mie resonances, which correspond to the magnetic dipole (MD) and electric dipole (ED) resonances and present circular displacement currents of electric and magnetic fields inside the particle, respectively [9]. By changing dielectric particles' geometrical parameters, MD and ED resonances can be tuned independently, facilitating the construction of diverse electromagnetic/optical devices, such as magnetic mirror [10], perfect reflector [11], quarter-wave and half-wave plates [12], and Huygens' metasurfaces with a near-unity transmission and a full phase coverage (0~2*π*) to reshape wavefront [13–17].

For almost all the practical applications, dielectric particles are placed on the substrate which might modify the far-field scattering and the near-field distribution of dielectric particle substantially. Previous studies have investigated the effect of various substrates such as SiO_2_, Ag, and Au and perfect electric conductor (PEC) on the scattering from dielectric particle [18–20]. Of special interest is the metallic substrate where the scattering spectrum will be significantly modified due to the coupling of Mie resonances with its mirror image. Compared with the isolated dielectric particle, dielectric particle placed on metallic substrate not only yields a more efficient far-field scattering but also a stronger near-field enhancement [21]. Up to now, by the theoretical analysis, numerical simulation, and experimental characterization, a series of novel phenomena have been discovered, such as magnetoelectric coupling between ED and MD resonances [18], broadband suppression of scattering [19], and mirror-image-induced magnetic modes [22]. However, the Mie resonant frequencies in these cases are influenced by various factors including material property, structure, and resonance mode and exhibit a complex variation with the gap size between dielectric particle and metallic substrate, which still needs a comprehensive mechanism interpretation. For example, the hybrid MD resonant wavelength of Si nanorod on Ag substrate demonstrates a blue shift as the gap size increases [21], while for the case of Si nanorod on Au substrate, the resonant wavelength is blue-shifted with the decrease of thin film thickness [23]. In addition, some researches demonstrate that the MD resonant wavelength is hardly changed or slightly red-shifted when Si nanosphere is away from metallic substrate [24, 25]. Although this problem was mainly reported in the near-infrared or visible regime, it is a universal phenomenon for microwave, terahertz, and other frequencies due to the mirror image principle. It is important to figure out the dependency relationship between resonant frequency and their gap size. Such a frequency shift will sharply degrade the performance of many optical devices designed at specific wavelength, such as nonlinear optics [23, 24, 26] and reflection-type metasurfaces [27–30].

In this work, we systematically study the effect of metallic substrate on the MD resonant frequency of dielectric resonators and elucidate the physical mechanisms. For a typical dielectric resonator of CaTiO_3_ ceramic block (sphere and spherical segment) on metallic substrate, the variation of MD resonant frequency is clarified, as well as the dominant mechanisms for different stages. Furthermore, based on the sharp frequency shifting on small gaps, the application of dielectric sensor is proposed for measuring either the thickness of ultrathin film or its dielectric constant, and a governing equation is derived from the method of symbolic regression.

## 2. Results and Discussions

### 2.1. MD Resonance of CaTiO_3_ Ceramic Block

**Figure 1:**
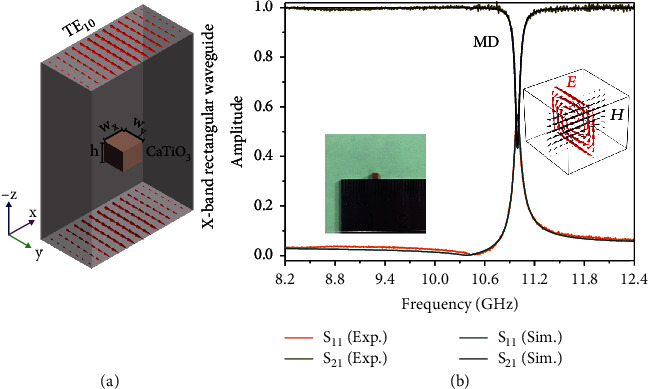
(a) Schematic of experimental setup. (b) The measured and simulated results for CaTiO_3_ ceramic block. The insets (left and right) are the sample and the field distribution at the MD resonance, respectively.

The proposed dielectric resonator is made of CaTiO_3_ ceramic which is doped with 1 wt.% ZrO_2_ and sintered for 2 h at 1350°C and then wire-cut into a rectangular block with *w*_*x*_ = *w*_*y*_ = 1.98 mm and *h* = 1.62 mm (left inset, Figure 1(b)) for exciting the Mie resonances at X-band. In order to accurately estimate the complex permittivity and figure out the resonance property of CaTiO_3_ ceramic block, we first use the metallic rectangle waveguide (WR90) to measure the transmission and reflection spectra (S_11_ and S_21_), where the ceramic block is placed in the center of waveguide and the short side (*h*) is along the direction of wave propagation (Figure 1(a)). As illustrated in Figure 1(b), the near-unity transmission S_21_ covers the whole region frequency range expect a sharp resonance at 11.00 GHz. Subsequently, we perform the finite-element simulation (COMSOL Multiphysics, RF module, spectral analysis) and match the simulated result with the experimental data, as shown in Figure 1(b), obtaining a permittivity of 160.5 − 0.056*i* for CaTiO_3_ ceramic. The distribution of electric field at this resonance is strongly confined inside the ceramic block and presents a closed loop, indicating a MD resonance (Figure 1(b), right inset).

### 2.2. Frequency Shifting of MD Resonance of Ceramic Block near Metallic Substrate

**Figure 2:**
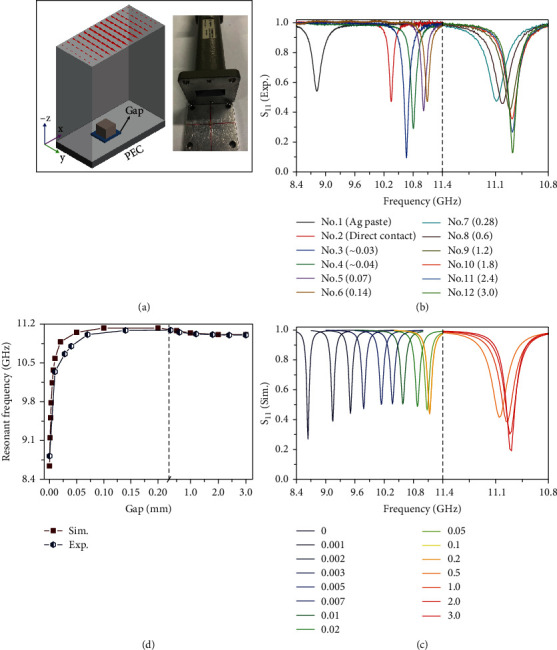
(a) Illustration of reflection-type setups. (b) Measured and (c) simulated reflection spectra for different gap sizes (in mm). (d) Plot of the resonant frequency as a function of gap size between dielectric block on metallic substrate.

To investigate the influence of metallic substrate on the MD resonance, we place the CaTiO_3_ ceramic block on the metallic surface and regulate their gap size primarily through low permittivity double-sided tapes (3 M 467MP, *ε* = 2.7) and cyanoacrylate instant adhesive (Loctite 498, *ε* = 2.3) in the following content.  Figure 2(a) displays the experimental apparatus where the size parameters and orientation of ceramic block are the same as Figure 1(a). Due to the existence of thick metallic substrate, the transmission spectra S_21_ are equal to zero across the entire frequency range. The reflection spectra S_11_ (Figure 2(b)) feature sharp dips stemming from the dielectric absorption at the metallic substrate-induced MD resonances. The difference in absorption/reflection amplitude is attributed to the tradeoff between dissipative loss rate and radiation rate which can be described by the critical coupling mechanism [31]. Compared to the change of absorption/reflection amplitude, we are more interested in the shifting of resonant frequency for different gap sizes. For large gaps between 0.07 and 3.0 mm (No. 5~No. 12), the height of dielectric spacer is varied by controlling the number of double-sided tape layers. As illustrated in Figure 2(b), the resonant frequencies are first increased and then decreased with increasing the gap size, but always in the vicinity of the MD resonance of the isolated ceramic block (11.00 ± 0.10 GHz). For relatively small gaps filled with the instant adhesive (No. 3 and No. 4), the resonance positions shift to lower frequencies (10.66 and 10.80 GHz). The reflection dips with different amplitudes are mainly attributed to the level of curing reaction of instant adhesive (Figure S1) [32]. Furthermore, we make adjacent two surfaces directly contact with each other (No. 2). Since the limitation of machining precision, the surface of ceramic block and metallic substrate are not smooth or even absolutely, resulting in an incomplete dielectric-metal contact and corresponding an effective gap size about 0.007 mm (concluded by the simulation in Figure 2(c)), so that the resonant frequency is red-shifted to 10.35 GHz. It is noted that although the dielectric spacer in this case is the vacuum whose permittivity is less than those of instant adhesive and double-sided tapes, the resonant frequency still demonstrates a red shift, thereby confirming that the red shift of resonant frequency is derived from the decrease of gap size other than the permittivity of dielectric spacer. To achieve the absolutely zero gap size, we use the conductive Ag paste to glue two surfaces together (No. 1), in which case the reflection dip appears at the lowest frequency of 8.82 GHz.

Finite-element simulations are performed to validate the above performance of frequency shifting with varying gap sizes where the maximum mesh size of dielectric spacer is smaller than a quarter of gap size in the propagation direction to guarantee the calculation accuracy. Since the big difference of permittivity between CaTiO_3_ ceramic and dielectric spacer (instant adhesive and double-sided tape), the resonant frequencies for big gap sizes are in proximity to each other. For simplicity and without loss of generality, we remove the dielectric spacer layer and model it as air in the following simulations. As illustrated in Figure 2(c), the resonant frequency of metallic structure-induced MD mode dramatically blue shifts with increasing gap size, from 8.64 GHz at gap = 0 mm to 11.10 GHz at gap = 0.2 mm. Interestingly, if the gap continues to increase from 0.2 to 3.0 mm, the resonance position is slightly red-shifted to a low frequency instead. By extracting the data from Figures 2(b) and 2(c), we plot the simulated and experimental resonant frequencies as a function of gap size, which are coincided with each other well (Figure 2(d)). A little difference is mainly attributed to the permittivity difference of dielectric spacer between simulations and experiments and measurement error of dielectric spacer thickness especially for tiny gaps. From an overall perspective, the resonant frequencies of metallic structure-induced MD mode present an exponential dependence of gap size (see Equations (2) and (3)). But from a local viewpoint, it also demonstrates a downtrend in a narrow-frequency bandwidth for large gaps (>0.2 mm).

**Figure 3:**
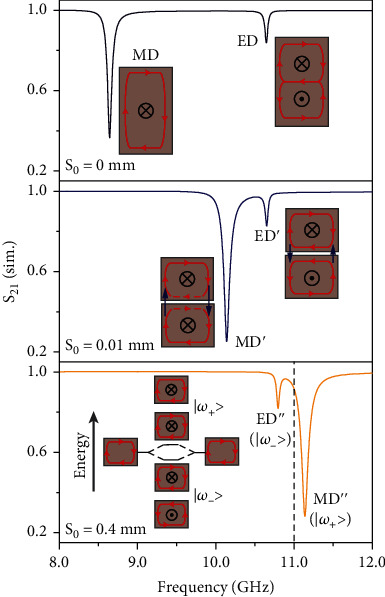
Simulated transmission spectra for different gap sizes of S_0_ = 0 (top), 0.01 mm (middle), and 0.4 mm (bottom). The insets show the field distribution at Mie resonances, where the red and blue arrows represent the displacement electric field in the dielectric and gap, respectively.

To elucidate the above electromagnetic response of dielectric on metal with varying gap size, it is instructive to investigate this configuration as a ceramic block together with its image in transmission mode [22]. The top panel of Figure 3 depicts the transmission spectrum for S_0_ = 2 × gap = 0 mm where two transmission dips are corresponding to the MD and ED resonances (insets in top panel of Figure 3), respectively. The resonant frequency of MD mode is equal to the case of half-height ceramic block on the metallic substrate (gap = 0, Figure 2(c)), while the ED mode with a symmetric electric field distribution, i.e., antisymmetric magnetic field distribution, does not exist in the case of ceramic block on metallic structure due to the parity of image coupling. Increasing the gap size S_0_ to 0.01 mm (middle panel, Figure 3), the resonant frequency of MD′ mode is blue-shifted dramatically, while the resonant frequency of ED′ mode is hardly changed. At the resonant frequencies of MD′ and ED′ modes, two out-plane magnetic dipoles in dielectric blocks are aligned parallel and antiparallel (insets in the middle panel of Figure 3), intuitively resulting in higher and lower resonant frequencies, respectively [33]. However, two greatly enhanced, vertical, and antiparallel electric fields (left inset in the middle panel of Figure 3) in the gap play a key role to determine the resonant frequency or energy of MD′ mode when the gap size is very small. As the gap is increased, the greatly enhanced electric field pair in the gap which contributes to the formation of electric displacement loops or magnetic dipole resonances in ceramic blocks is decreased exponentially (Figure S2), increasing the resonant frequency or energy of MD′ mode. For the ED′ mode, the electric field distribution in dielectric resonator is almost identical with the ED mode at S_0_ = 0 mm. Although two vertical and antiparallel electric fields in the gap are enhanced, the overall effect on the formation of electric displacement loops are cancelled out due to the opposite circumferential directions in upper and lower dielectric blocks (right inset in the middle panel of Figure 3), resulting in a hardly changed resonant frequency. For a large gap size S_0_ = 0.4 mm, the enhancement of vertical electric field pair in the gap can be neglected (Figure S2), and the coupling of two MD resonances plays a key role and leads to the spectral splitting (insets in the bottom panel of Figure 3) [33]. For the ED^″^ mode, the north and south poles of the two neighboring magnetic dipoles attract each other and therefore lead to the lower resonance frequency (|*ω*_−_〉), while for the MD^″^ mode, the poles with same sign are repulsive, which results in a higher resonance frequency (|*ω*_+_〉). As the gap is increased (>0.2 mm), MD^″^ and ED^″^ modes are red-shifted and blue-shifted, respectively, and approximate to the resonant frequency of the single dielectric block. As a whole, the resonant frequency of metallic substrate-induced MD mode mainly depends on the greatly enhanced electric field pair for small gaps; the coupling of MD resonance in the dielectric and its mirror image is responsible for large gaps; in the vicinity of the critical gap size, the tradeoff between the enhanced electric field in gap and the coupling of two MD resonances will determine the metallic substrate-induced resonant frequency.

A further study shows that the existence of metallic substrate not only affects the frequency of MD resonance but also the ED resonance which blue shifts with increasing gap size overall (Figure S3).

### 2.3. Frequency Shifting of MD Resonance of Ceramic Sphere near Metallic Surface

**Figure 4:**
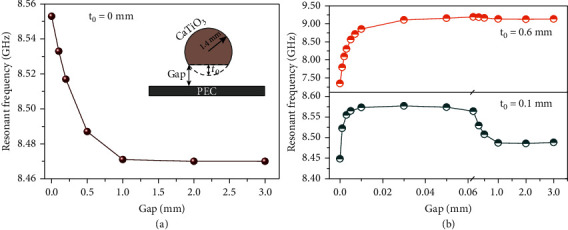
Plot of the resonant frequency as a function of gap size for dielectric (a) sphere and (b) spherical segments on metallic surface. The inset in (a) shows the cross-section of simulated unit cell.

In contrast to the dielectric block, high-refractive-index dielectric spheres also have been extensively studied, both experimentally and theoretically. Since the electromagnetic field is strongly confined in dielectric particles at Mie resonances, the in-plane coupling can be neglected for dielectric sphere array with large periodicities that their electromagnetic responses are similar with the case of isolated dielectric sphere. Therefore, we employ the unit cell with periodic boundary conditions to investigate the effect of varying gap size between ceramic sphere and metallic substrate on the MD resonance (Figure 4(a)) for a wider operating frequency range.

For the CaTiO_3_ ceramic sphere directly placed on metallic substrate (*t*_0_ = gap = 0 mm), the allowable dielectric-electric connectivity is only a single point that the region with greatly enhanced electric field pair in the gap is limited and cannot play the decisive role in resonant frequency. Conversely, increasing the gap size will weaken the coupling effect of two MD resonances in the dielectric and its mirror image, resulting in a red shift of resonant frequency (Figure 4(a)). On the whole, compared with the ceramic block, the MD resonant frequency of ceramic sphere is not sensitive to the metallic substrate and only fluctuates in a narrow bandwidth.

In practice, owing to the own gravity and machining precision, the point contact of dielectric sphere on metallic substrate is usually transformed into a surface contact (i.e., spherical segment). Figure 4(b) shows the resonant frequency of substrate-induced MD mode as a function of gap size for different CaTiO_3_ spherical segments. As *t*_0_ is increased, the allowable contact area becomes bigger that the greatly enhanced electric field pair will dominate the resonant frequency for small gaps. Therefore, the resonant frequency of metallic structure-induced MD mode for spherical segments demonstrates a similar behavior with dielectric block on metallic substrate, i.e., a blue shift first and then a red shift with increasing gap size.

### 2.4. Thin Film Thickness and Permittivity Measurements

From the above studies, we can conclude that for dielectric particle with a large contact area, the smaller gap size, the more pronounced frequency shifting of metallic substrate-induced MD resonance. Taking advantage of this property, we can achieve ultrasensitive close-range or dielectric film thickness detection. The sensitivity defined as resonant frequency shift per the gap size or dielectric film thickness change unit (*S* = |Δ*f*_*MD*_|/|Δ*gap*|) is employed to evaluate its sensing capability. For example, in the measurement of CaTiO_3_ ceramic block near metallic surface, when the thickness of instant adhesive is increased from 0.03 to 0.04 mm, the experimental resonant frequency is blue-shifted from 10.66 to 10.80 GHz, corresponding a sensitivity of 14.0 GHz/mm. Assuming the measurement accuracy is 1 MHz (a very conservative choice), the thickness resolution can achieve 71 nm.

To better demonstrate its ultrasensitive sensing capability to small gaps and promising application in measuring dielectric property, we conduct a proof-of-concept demonstration that uses the ceramic block to estimate the permittivity and thickness of dielectric thin film coated on the surface of good conductor. As the localized field of dielectric resonator is very sensitive to the change of dielectric environment contained in the deep-subwavelength gap region, the metallic substrate-induced MD resonant frequency can be expressed as a nonexplicit function of permittivity and thickness of dielectric thin film

(1) $$\begin{array}{c} f\left(\varepsilon ,t\right)=f_{MD}. \end{array}$$

**Figure 5:**
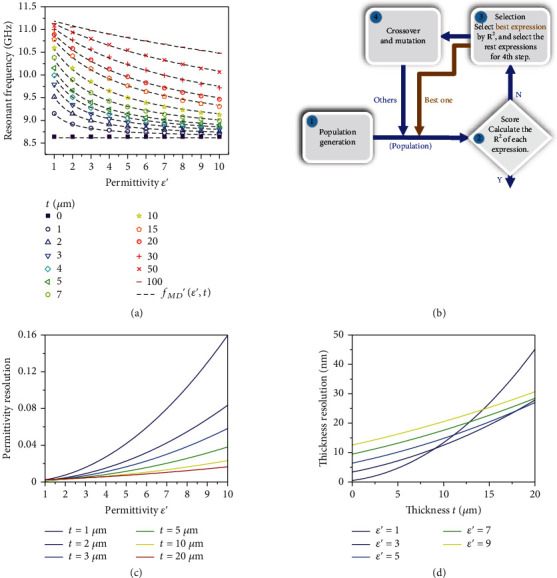
(a) Simulated resonant frequencies for dielectric films with varying permittivity and thickness. The dash lines are fitted by the method of symbolic regression. (b) Symbolic regression flow. (c) Permittivity resolution and (d) thickness resolution as a function of permittivity and thickness.

To determine Equation (1), we first perform the eigenmode analysis (COMSOL Multiphysics, RF module) and get the complex eigenfrequencies *f*_*MD*_ = *f*_*MD*_′ + if_*MD*_′′ for the dielectric thin film with varying thickness and permittivity and then use the method of symbolic regression (SR, a genetic programming algorithm) to predict their functional relationship based on the simulated data [34–36]. Since the dielectric loss of thin film only affects the imaginary part of complex frequency (*f*_*MD*_′′), we just focus on the dependence of real part of complex frequency (*f*_*MD*_′) on the thickness (*t* = 0 ~ 0.1 mm) and the real part of complex permittivity (*ε*′ = 1 ~ 10), which are depicted as the symbols in Figure 5(a). It can be seen that the metallic substrate-induced MD resonant frequency is decreased with the increase of permittivity, and the rate of change of resonant frequency tends to a constant with the increase of thickness. To accurately characterize the change law (Equation (1)), the method of SR is adopted and implemented by the binding genetic programming (BGP) package [37]. Figure 5(b) shows the SR flow where the first population is formed by 1000 expressions, and each of them is generated by randomly combining two descriptors (*t* and *ε*′) [38, 39]. Then, these expressions are evaluated by the fitness function of *R*^2^ and selected by the so-called tournament method [40]. The best one that survives is used in the next generation of population directly. For the residual expressions, crossover and mutations are employed to generate new expressions which combine with the expressions that get through to form the next generation of population. This iteration process is repeated until the *R*^2^ of the best target-unit expression is unchanged for 20 generations. Eventually, the prediction model of metallic substrate-induced MD resonant frequency (governing equation) is found to be in the following form:

(2) $$\begin{array}{c} {f_{MD}}^{\prime }=6.589e^{\left(0.115t_{*}/0.00116\varepsilon^{\prime }+0.329t_{*}\right)-0.101t_{*}}+2.031\;\left(\text{GHz}\right), \end{array}$$

where *t*_∗_ = *t*/*t*_*c*_ (*t*_*c*_ = 1 mm) is the nondimensional thickness of dielectric film. It is observed that the dashed lines calculated by Equation (2) agree with the simulated results well (Figure 4(a)). Therefore, according to the measured resonant frequency and the known thickness, we can retrieve the permittivity of dielectric film based on Equation (2). Similarly, we can use the ceramic resonator to nondestructively detect the thickness through the known permittivity and measured resonant frequency, without breaking the sample for a cross-sectional observation in the scanning electron microscope. Furthermore, to alleviate the coupling of two MD resonances and better clarify the effect of greatly enhanced electric field pair on the resonant frequency, we limit the variation of thin film thickness to a small range (0 ≤ *t* ≤ 5 *μm*); the governing equation performed by symbolic regression can be expressed as

(3) $$\begin{array}{c} {f_{MD}}^{\prime }=10.61-1.96e^{-289.70t_{*}/\varepsilon \prime }\left(GHz\right). \end{array}$$

Obviously, the metallic-induced MD resonant frequency presents an exponential relationship with the ratio of thickness to permittivity.

For quantitatively characterizing its sensing capability, we conduct the partial derivative of *f*_*MD*_′ with respect to *t* and *ε*′ based on Equation (2). By assuming the measurement accuracy of 1 MHz, the permittivity resolution and thickness resolution are calculated. Figure 5(c) shows the permittivity resolution which is decreased with the increase of permittivity but increased with the thickness. As for the thickness resolution, it is decreased with the increase of thickness, and the rate of change of thickness resolution is higher for dielectric film with smaller permittivity and bigger thickness (Figure 5(d)). Nevertheless, taken as a whole, the permittivity resolution is superior to 1.6% of thin film permittivity, and the thickness measurement can reach a nanometer level resolution.

## 3. Conclusions

In summary, a high permittivity CaTiO_3_ ceramic block near metallic substrate is proposed to study the effect of gap size on the MD resonant frequency by experiments and simulations. We find that there are two physical mechanisms to codetermine the metallic substrate-induced MD resonance, resulting in different frequency shifting for different stages. As gap size increases, the metallic substrate-induced MD resonant frequency is dominated by the greatly enhanced, vertical, and antiparallel electric field pair in the gap and the coupling of MD resonance in the dielectric and its mirror image, respectively, leading to an exponential blue shift first and then slight red shift. According to the proposed two mechanisms, we can give a reasonable explanation that ceramic sphere on metallic substrate demonstrates different frequency shifting with varying gap size. Furthermore, since the MD resonant frequency is ultrasensitive to metallic substrate with small gaps, the ceramic block is served as a dielectric sensor using for the accurate detection of thin film thickness or permittivity by the method of symbolic regression. We believe our findings will provide a more comprehensive understanding of frequency shifting of metallic substrate-induced MD resonance with varying gap size and facilitate to the design of ultrasensitive sensors, nonlinear applications, or other reflection-type metasurfaces.

## Data Availability

Data supporting the findings of this study are available in the main text or the supplementary information.

## Supplementary Materials

Supplementary Materials Figure S1: reflection spectra of CaTiO_3_ ceramic block on metallic substrate with a dielectric spacer of instant adhesive at different reaction times. Figure S2: the ratio between the maximum vertical electric field in the gap and incident electric field as a function of gap size. Figure S3: (a) the measured and simulated results for CaTiO_3_ ceramic block with *w*_*x*_ = *w*_*y*_ = 2.0 mm and *h* = 2.2 mm. (b) The measured and simulated reflection spectra of dielectric on metallic substrate with different gap sizes.
