# Supplementary material for: Ultrasensitive Frequency Shifting of Dielectric Mie Resonance near Metallic Substrate
Source: Research (Wash D C). 2022 May 9;2022:9862974. doi: 10.34133/2022/9862974 (PMC9115667; doi:10.34133/2022/9862974)
Supplement: Supplementary Materials — Figure S1: reflection spectra of CaTiO3 ceramic block on metallic substrate with a dielectric spacer of instant adhesive at different reaction times. Figure S2: the ratio between the maximum vertical electric field in the gap and incident electric field as a function of gap size. Figure S3: (a) the measured and simulated results for CaTiO3 ceramic block with wx = wy = 2.0 mm and h = 2.2 mm. (b) The measured and simulated reflection spectra of dielectric on metallic substrate with different gap sizes. [file 9862974.f1.docx]

Supplementary Materials for

**Ultrasensitive Frequency Shifting of Dielectric Mie Resonance near Metallic Substrate**

Chuanbao Liu^1^, Changxin Wang^2^, Junhong Chen^1^, Yanjing Su^2^, Lijie Qiao^2^, Ji Zhou^3^ and Yang Bai^2*^

*Corresponding author. Email: [baiy@mater.ustb.edu.cn](mailto:baiy@mater.ustb.edu.cn)

**This PDF file includes:**

Fig. S1. Reflection spectra of CaTiO_3_ ceramic block on metallic substrate with a dielectric spacer of instant adhesive at different reaction times.

Fig. S2. The ratio between the maximum vertical electric field in the gap and incident electric field as a function of gap size.

Fig. S3. (a) The measured and simulated results for CaTiO_3_ ceramic block with *w_x_* = *w_y_* = 2.0 mm and *h* = 2.2 mm. (b) The measured and simulated reflection spectra of dielectric on metallic substrate with different gap sizes.


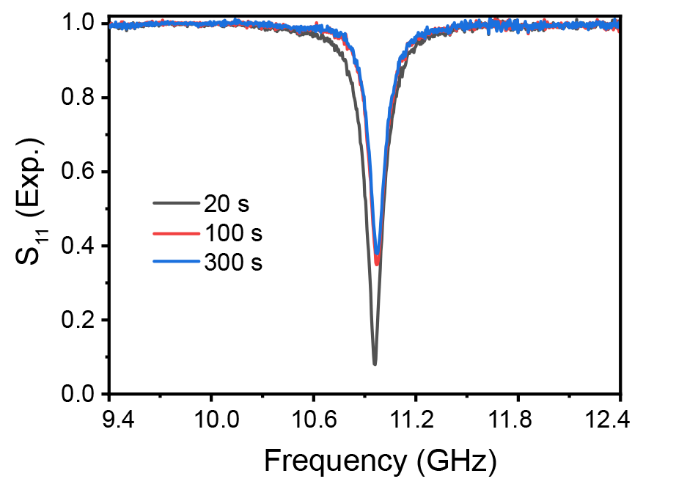


**Fig. S1**. Reflection spectra of CaTiO_3_ ceramic block on metallic substrate with a dielectric spacer of instant adhesive at different reaction times.


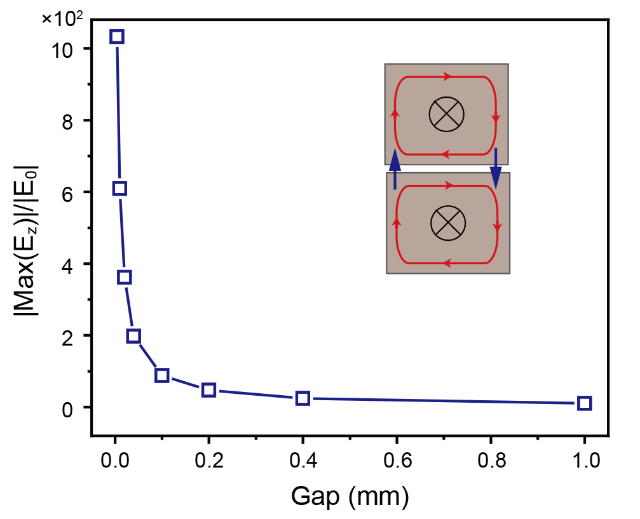


**Fig. S2.** Plot of the ratio between the maximum vertical electric field in the gap and incident electric field as a function of gap size.


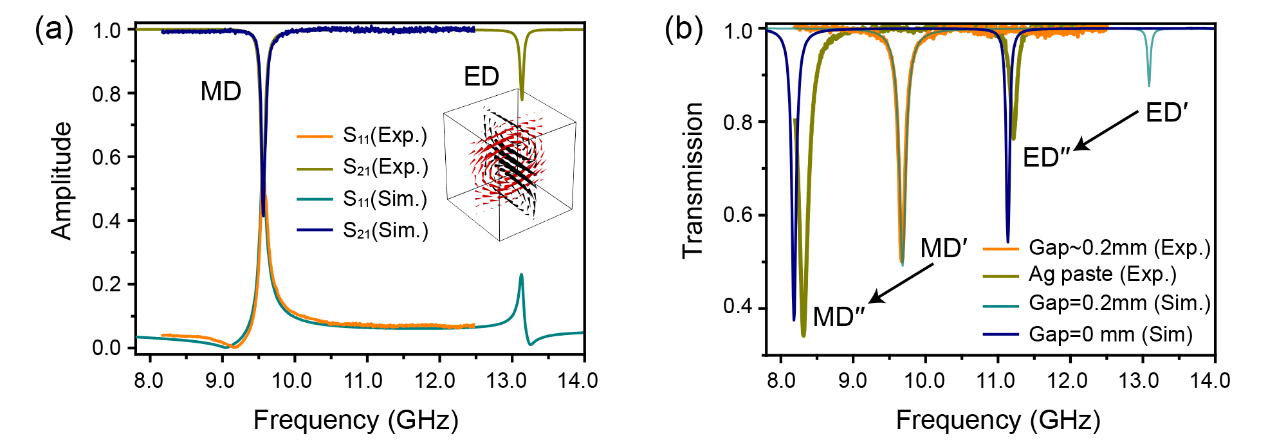


**Fig. S3.** (a) The measured and simulated results for CaTiO_3_ ceramic block with *w_x_* = *w_y_* = 2.0 mm and *h* = 2.2 mm. (b) The measured and simulated reflection spectra of dielectric on metallic substrate with different gap sizes.
